# The Snail Transcription Factor Regulates the Numbers of Neural Precursor Cells and Newborn Neurons throughout Mammalian Life

**DOI:** 10.1371/journal.pone.0104767

**Published:** 2014-08-19

**Authors:** Mark A. Zander, Gonzalo I. Cancino, Thomas Gridley, David R. Kaplan, Freda D. Miller

**Affiliations:** 1 Program in Neuroscience and Mental Health, Hospital for Sick Children, Toronto, Ontario, Canada; 2 Institute for Medical Sciences, University of Toronto, Toronto, Ontario, Canada; 3 Department of Molecular Genetics, University of Toronto, Toronto, Ontario, Canada; 4 Department of Physiology, University of Toronto, Toronto, Ontario, Canada; 5 Maine Medical Center Research Institute, University of Maine, Scarborough, Maine, United States of America; Universitätsklinikum Carl Gustav Carus an der Technischen Universität Dresden, Germany

## Abstract

The Snail transcription factor regulates diverse aspects of stem cell biology in organisms ranging from Drosophila to mammals. Here we have asked whether it regulates the biology of neural precursor cells (NPCs) in the forebrain of postnatal and adult mice, taking advantage of a mouse containing a floxed *Snail* allele (*Snail^fl/fl^* mice). We show that when *Snail* is inducibly ablated in the embryonic cortex, this has long-term consequences for cortical organization. In particular, when *Snail^fl/fl^* mice are crossed to Nestin-cre mice that express Cre recombinase in embryonic neural precursors, this causes inducible ablation of *Snail* expression throughout the postnatal cortex. This loss of Snail causes a decrease in proliferation of neonatal cortical neural precursors and mislocalization and misspecification of cortical neurons. Moreover, these precursor phenotypes persist into adulthood. Adult neural precursor cell proliferation is decreased in the forebrain subventricular zone and in the hippocampal dentate gyrus, and this is coincident with a decrease in the number of adult-born olfactory and hippocampal neurons. Thus, Snail is a key regulator of the numbers of neural precursors and newborn neurons throughout life.

## Introduction

Radial neural precursor cells of the developing cortex must proliferate, differentiate and in some cases, undergo apoptosis in order to populate the postnatal cortex with the appropriate numbers and types of cells to generate cortical neural circuitry [1]. While most of these multipotent precursor cells ultimately differentiate into neurons and glia, a subset fulfill the criteria for bona fide stem cells, since they persist as a subpopulation of neural stem cells in the adult forebrain subventricular zone (SVZ) niche [2]. In this regard, we are just now starting to understand the molecular mechanisms that regulate the selection and maintenance of those cortical neural stem cells that persist from the embryo into adulthood. However, it is clear that at least some regulatory mechanisms are important for determining the biology of these forebrain precursors throughout life, as we have previously shown for the CBP histone acetyltransferase [3]– and the p53 family [6]–[9]. Moreover, recent evidence indicates that perturbations that affect embryonic cortical precursors can have long-lasting effects on the adult brain by regulating the size and characteristics of the adult neural stem cell pool. For example, we recently showed that IL-6 regulates the self-renewal and thus numbers of embryonic cortical precursors, and in so doing determines the size of the adult forebrain neural stem cell pool [10].

How then might we define the molecular mechanisms that regulate the proliferation and maintenance of cortical radial precursor cells throughout life? Intriguingly, a number of recent reports suggest that the molecular factors controlling the behavior of radial precursors are, in part, conserved between *Drosophila* and mammals [11]–[14]. In this regard, one key regulator of neural precursor biology in model organisms is the Snail transcription factor. Snail, a zinc-finger transcription factor, has been shown to control both transcriptional repression and activation [15]. In Drosophila, Snail and its closely related family members Escargot and Worniu control neuroblast (NB) proliferation through regulation of cell cycle genes, one of which is the cdc25 phosphatase orthologue *string* [16]–[18]. Additionally, work in *C. elegans* has revealed a role for the Snail orthologue CES-1, in regulating a BH3-only apoptotic pathway in the neurosecretory motoneuron (NSM) cell lineage after an asymmetric cell division [19], [20].

Recent work indicates that Snail and its family members play a similar role in the embryonic cortex. In particular, we recently showed that Snail regulates embryonic radial precursor survival and proliferation via regulation of two distinct targets, p53 and cdc25b [21], and another recent publication [22] showed that Scratch, a Snail superfamily member, regulates delamination of newborn cortical neurons from the cortical apical epithelium by transcriptionally repressing downstream targets like the neurogenins and E-cadherin. However, while these studies indicate important roles for the Snail superfamily in the embryonic cortex, it is still unclear whether Snail regulates that subpopulation of radial precursors that persist into postnatal life and/or whether it regulates adult neural stem cells themselves.

Here, we have asked whether Snail also regulates postnatal and adult forebrain neural precursors, taking advantage of a mouse line carrying a floxed *Snail* allele. We confirm that in the embryonic cortex Snail regulates radial precursor numbers and proliferation, as well as neuronal localization. Moreover, we show that inducible ablation of *Snail* in embryonic precursors and all of their progeny in the postnatal cortex leads to postnatal precursor and neuronal deficits, and decreases in adult neural precursor cells (NPCs) and adult neurogenesis.

## Materials and Experimental Procedures

### Animals

All animal use was approved by the Animal Care Committee of the Hospital for Sick Children (Protocol #28136) in accordance with the Canadian Council of Animal Care policies. *Snail^fl/fl^* mice [23] and *Nestin-cre* mice [24] were genotyped and maintained as described in [23] and [24] respectively. To obtain *Snail^fl/fl^;nestin-cre* and *Snail^fl/fl^* littermates for analysis, *Snail^fl/fl^;nestin-cre* males were bred with *Snail^fl/fl^* females. The *Snail^fl/fl^* mice are on a 129S1/SvImJ background, and the *Nestin-cre* mice on a CD1 background. CO2 gas was used to humanely euthanize mice in this study.

### Plasmids

The Cre recombinase overexpression vector pCIG2-Cre-IRES-EGFP and negative control plasmid pCIG2-IRES-EGFP were kindly provided by the laboratories of Dr. Francois Guillemot [25] and Dr. Ulrich Mueller [26], respectively.

### *In utero* electroporation

*In utero* electroporation was performed as described [27] with *Snail^fl/fl^* mice, injecting a total of 4 µg of plasmid DNA and 0.5% trypan blue as a color indicator for successful injection of plasmid DNA. The square electroporator CUY21 EDIT (TR Tech, Japan) was used to deliver five 50 ms pulses of 40–50 V with 950 ms intervals per embryo. Brains were dissected 3 days post transfection in ice-cold HBSS, fixed in 4% paraformaldehyde at 4°C overnight, cryopreserved and cryosectioned coronally at 16 µm.

### Immunocytochemistry and histological analysis

Immunocytochemistry on cryosections was performed as previously described [21], [28], except for immunostaining for Snail. The primary antibodies used were rabbit anti-GFP (1∶5000; Abcam), chicken anti-GFP (1∶1000; Abcam), rabbit anti-Tbr1 (1∶250; Abam), rabbit anti-Pax6 (1∶1000; Covance), rabbit anti-Tbr2 (1∶250; Abcam), mouse anti-Satb2 (1∶400; Abcam), rabbit anti-cleaved caspase 3 (1∶200; Millipore), mouse anti-Ki67 (1∶200; BD Biosciences), goat anti-Snail-53519 (1∶1000 for paraffin sections; Abcam), rabbit anti-BrdU (1∶200; Accurate Chemical), goat anti-Sox2 (1∶500; Santa Cruz), goat anti-DCX (1∶200; Santa Cruz), and mouse anti-NeuN (1∶500; Millipore). The secondary antibodies used were Alexa Fluor 555-, Alexa Fluor 488- and Alexa Fluor 647- conjugated goat/rat antibodies to mouse, rabbit, goat, chicken IgG (1∶1000 for 488/555, 1∶500 for 647; Invitrogen). For Snail immunostaining, embryonic brains were formalin fixed and paraffin embedded, and after cutting, sections were de-paraffinized with xylene and rehydrated with an ethanol gradient. Antigen retrieval was performed with 10 mM sodium citrate buffer (pH 6) with 0.05% Tween 20 at 95°C for 20 minutes. Sections were blocked at room temperature with 4% donkey serum in PBS, and incubated with goat anti-Snail 53519 (1∶1000; Abcam) overnight at 4°C. Sections were washed with PBS containing 0.2% Tween 20 and detected using donkey anti-goat Alexa Fluor 555 at 1∶1000 in PBS. Nuclei were stained with Hoechst 33258 (Sigma). BrdU staining was performed on embryonic brains that were fixed in 4% PFA overnight followed by cryopreservation in 30% sucrose overnight.

### Analysis of post-natal neural precursors and neurons

For postnatal cortical analyses pregnant *snail^fl/fl^* mice that had been bred to *snail^fl/fl^;nestin-cre* male mice were injected with BrdU (Sigma) dissolved in PBS at a dose of 100 mg per kg of body weight at gestational day 13. Cortices were sectioned coronally at 18 µm. For BrdU double labeling, sections were first immunohistochemically stained as previously mentioned [21], [28], and incubated in one of goat anti-Sox2, mouse anti-Ki67, rabbit anti-Tbr1, or mouse anti-Satb2 primary antibodies overnight at 4°C, followed by immunostaining in donkey anti-goat, -mouse, or -rabbit Alexa 488 secondary antibodies, respectively. These sections were then fixed for 15 minutes in 4% PFA, washed in 1X PBS, and incubated in 0.5 N HCL for 30 min at 60°C and washed in 1X PBS buffer for 5 min before blocking as usual. Primary anti-BrdU antibody was then incubated overnight at 4°C, followed by immunostaining with donkey anti-rat Alexa 555 secondary antibody. Double labeled cells were then quantified from 3–4 anatomically matched sections spanning the SVZ and cortex from cre-positive and –negative brains.

### Analysis of adult neural precursors and adult-born neurons

For adult SVZ and SGZ studies, adult mice were injected once with 100 mg/Kg BrdU and analyzed the next day. Brains were postfixed overnight and the SVZ and SGZ were sectioned coronally on a cryostat at 18 µm. Ten SVZ or dentate gyrus sections, sampled every tenth section, were analyzed immunocytochemically for BrdU as described [8]. For the SVZ and dentate gyrus, we quantified all BrdU-labelled nuclei. To perform the Sox2/BrdU analysis, sections were incubated in 1 N HCl at 60°C for 30 min, rinsed in PBS, incubated in rat anti-BrdU antibody at 4°C overnight, and then in Alexa 488 donkey anti-rat antibody for 2 hours followed by sequential immunostaining with anti-Sox2 followed by an Alexa Fluor 555-conjugated donkey anti-mouse secondary antibody. The total numbers of Sox2-positive, BrdU-positive, and double-labelled Sox2/BrdU-positive cells were then quantified in 10 sections through the SVZ or dentate gyrus, sampled every ten serial sections. To quantify adult-born hippocampal or olfactory bulb neurons, one month old mice were injected with 60 mg/kg BrdU intraperitoneally every 3 hours for five injections. Thirty days later, the hippocampi or olfactory bulbs were cryosectioned serially at 18 µm throughout their rostrocaudal extent. Every tenth serial section was immunostained, for a total of 10 sections per brain. To perform immunostaining, sections were incubated in 1 N HCl at 60°C for 30 min, rinsed in PBS, incubated in rat anti-BrdU antibody at 4°C overnight, and then in Alexa 488 donkey anti-rat antibody for 2 hours followed by sequential immunostaining with anti-NeuN followed by an Alexa Fluor 555-conjugated donkey anti-mouse antibody. The total numbers of BrdU-NeuN double labeled cells in the granule cell layers were counted, and to obtain the total relative number of BrdU-positive adult-born neurons, these numbers were multiplied by ten to account for sampling frequency.

### RT-PCR

Genotyping of mouse colonies was performed using Ampliqon taq DNA polymerase red (Denmark) with the following primers and PCR programs. Primers for genotyping the control floxed *Snail* allele were FloxF2 (5′-CGGGCTTAGGTGTTTTCAGA-3′) and FloxR (5′-CTTGCTTGGTACCTGCCTTC-3′); 94°C 1 min., (94°C 15 sec., 58°C 15 sec., 72°C 30 sec.) X 39 cycles, 72°C 5 min. Recombined *Snail* allele primers were FloxF2 and FloxDelR (5′-TGAAAGCGGCTCTGTTCAGT-3′); 94°C 1 min., (94°C 15 sec., 63°C 30 sec., 72°C 40 sec.) X 39 cycles, 72°C 5 min. Primers for genotyping *nestin-cre* mice were forward (5′-GCGGTCTGGCAGTAAAAACTATC-3′) and reverse (5′-GTGAAACAGCATTGCTGTCACTT-3′); 94°C 1 min., (94°C 10 sec., 60°C 10 sec., 72°C 3 min.) X 39 cycles, 72°C 5 min.

### Microscopy and quantification of cortical sections

For the embryonic studies, cortical sections were chosen that showed a similar anatomical distribution of EGFP-positive cells for comparison. Brains were sectioned at 16 µm at E17/18, and three to four brain sections at the same anatomical level per embryo were analyzed. Adult, post-natal and embryonic brain sections were imaged using an Olympus IX81 inverted fluorescence microscope equipped with a Hamamatsu C9100-13 back-thinned EM-CCD camera and Okogawa CSU X1 spinning disk confocal scan head. Stitching of 20X objective images was performed using Volocity (Perkin Elmer) software to cover the ventricular zone, SVZ and cortical plate of each coronal section. Ventricular, subventricular and cortical plate layers were delineated using Hoechst staining. For quantification of total cells per section, total EGFP- or BrdU-positive cells were counted in these stitched images to obtain total number of electroporated or BrdU labeled cells/section, respectively. For quantification of total electroporated cells that were positive for a given marker, the proportion of EGFP-positive cells expressing that marker was determined from quantification of a column spanning the ventricle to the meninges within the same sections, and this proportion was multiplied by the total number of EGFP-positive cells/section to obtain the total number of marker-positive cells. For quantification of double labeled BrdU-positive cells, a column spanning the ventricle to the meninges within the same section was counted from anatomically matched sections.

### Statistics

All data were expressed as the mean plus or minus the standard error of the mean (SEM), and were tested for statistical significance with Student's *t*-tests unless otherwise indicated, in which case they were analyzed with a Student Neuman-Keuls post-hoc ANOVA. Differences were considered significant if p<0.05.

All tests were performed using Prism 5 (GraphPad, La Jolla, CA).

## Results

### Acute genetic ablation of Snail in Snail^fl/fl^ cortices causes a decrease in embryonic cortical precursors and their neuronal progeny

**Figure 1:**
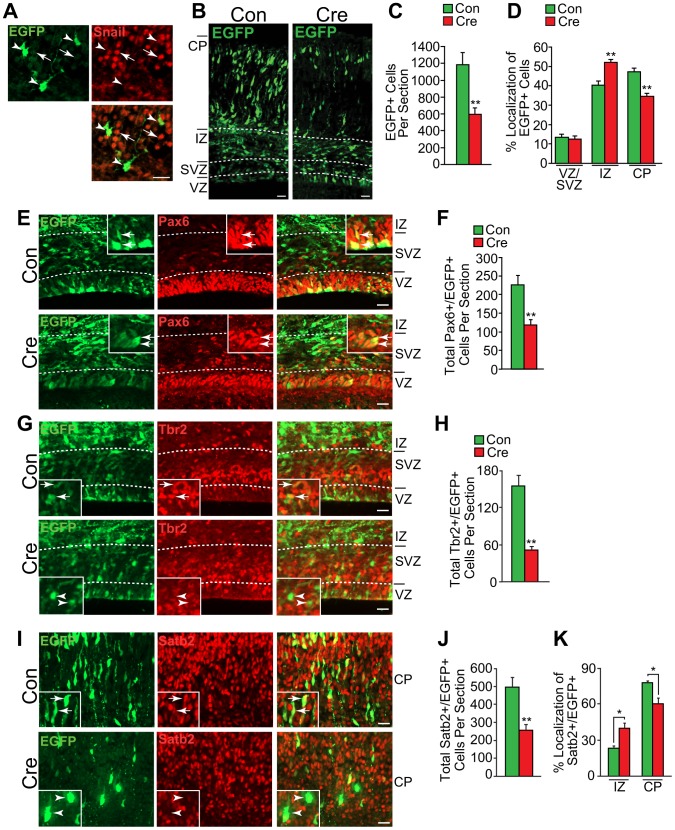
Acute Snail knockout decreases the numbers of neural precursors and neurons in the embryonic cortex. Cortices of *Snail^fl/fl^* mice were electroporated at E13/14 with an expression construct that encodes both Cre recombinase and EGFP (Cre), and were analyzed three days later. As a control, cortices were electroporated with the same construct that encodes EGFP alone (Con). (A) Confocal fluorescence micrographs of the cortical plate from Cre-EGFP electroporated brains stained with EGFP (green) and Snail (Red; bottom panel shows the merge). Arrows indicate EGFP-negative cells with robust nuclear Snail immunoreactivity, and arrowheads denote EGFP-positive cells with decreased Snail staining. Scale bar 20 µm. (B) Fluorescence micrographs of coronal sections through E16/17 control or Cre-electroporated cortices immunostained for EGFP (green). The boundaries between the different cortical regions are denoted by the dotted white lines. Scale bar, 20 µm. (C,D) Quantification of sections similar to those in B for the total number of EGFP-positive cells per section (C) and the relative location of EGFP-positive cells in the different cortical regions (D). (**p<0.01; n = 4 embryos each). (E) Confocal micrographs of coronal sections through the VZ/SVZ of E16/17 control or cre-electroporated cortices immunostained for EGFP (green) and Pax6 (red, right panels show the merges). The boxed insets show EGFP-positive cells at higher magnification. Arrows denote double-labeled cells, and the white hatched lines denote the borders of the different cortical regions. Scale bar, 20 µm. (F) Quantification of sections as in E for total Pax6-positive, EGFP-positive cells per section (F). (**p<0.01; n = 4 brains each). (G) Confocal micrographs of coronal sections through the VZ/SVZ of E16/17 control or Cre-electroporated cortices immunostained for EGFP (green) and Tbr2 (red, right panels show the merges). The boxed insets show EGFP-positive cells at higher magnification. Arrows denote double-labeled cells, and the white hatched lines denote the borders of the different cortical regions. Scale bar, 20 µm. (H) Quantification of sections as in G for total Tbr2-positive, EGFP-positive cells per section. (**p<0.01; n = 4 brains each). (I) Confocal micrographs of the cortical plate of E16/17 control or Cre-electroporated cortices immunostained for EGFP (green) and Satb2 (red, right panels show the merges). The boxed insets show EGFP-positive cells at higher magnification. Arrows denote double-labeled cells and arrowheads EGFP cells negative for Satb2. Scale bar, 20 µm. (J,K) Quantification of sections as in I for total Satb2-positive, EGFP-positive cells per section (J) and their relative localization in the intermediate zone (IZ) and the cortical plate (K). (**p<0.01; n = 4 brains each).

We recently demonstrated that acute shRNA-mediated knockdown of Snail in embryonic cortical precursors led to a decrease in numbers of neural precursors and newborn neurons that was due to increased apoptosis and decreased proliferation [20]. We therefore first confirmed that acute ablation of the Snail gene in a conditional knockout mouse model of Snail (*Snail^fl/fl^*) would recapitulate these same phenotypes. To do this, we *in utero* electroporated the cortex of E13/14 *Snail^fl/fl^* embryos with a plasmid expressing Cre-recombinase and EGFP from the same cistron [25]. As controls, *Snail^fl/fl^* embryos were electroporated with a similar plasmid lacking Cre recombinase [26]. At this age, *in utero* electroporation transfects cortical radial precursor cells that line the ventricles, and over the ensuing three days many of these cells will self-renew as radial precursors, while others will differentiate either into the neurogenic transit-amplifying cells in this system, intermediate progenitors, or directly into neurons [29]. To ensure that this caused a decrease in Snail expression in the electroporated cells, we immunostained coronal sections through the cortices of these embryos 3 days post-electroporation for EGFP and for Snail, using an antibody we have previously validated [21]. This analysis (Figure 1A) demonstrated that while almost all cells in the embryonic cortex were positive for nuclear-localized Snail, in the large majority of EGFP-positive cells, Snail immunoreactivity was decreased or undetectable.

Having ascertained that this approach was efficacious, we quantified the number and location of EGFP-positive cells within these electroporated cortices. This analysis revealed that cortices electroporated with the Cre-EGFP plasmid contained roughly half as many EGFP-positive cells as did those electroporated with the control EGFP plasmid (Figure 1B,C). Moreover, these cells were mislocalized relative to controls, with proportionately more EGFP-positive cells in the intermediate zone and fewer in the cortical plate region, which contains newborn neurons (Figure 1D).

To ask if precursors were affected by Cre-mediated recombination of the floxed Snail locus, we immunostained similar sections for EGFP and the radial glial precursor marker Pax6 (Figure 1E) or the intermediate progenitor marker Tbr2 (Figure 1G). In cortices electroporated with Cre-EGFP, there was a roughly two-fold decrease in the total number of EGFP-positive radial precursors cells (Figure 1F) and a four-fold decrease in the number of EGFP-positive intermediate progenitors (Figure 1H) relative to *snail^fl/fl^* cortices electroporated with EGFP alone. We also asked if neurons were affected by immunostaining similar sections for Satb2 (Figure 1I), a marker of layer 2–4 neurons that are targeted during the developmental window of our *in utero* electroporations [30]. Sections electroporated with the Cre-EGFP plasmid had significantly fewer EGFP-positive, Satb2-positive neurons relative to controls (Figure 1J), and these neurons were mislocalized, with proportionately more in the intermediate zone and fewer in the cortical plate (Figure 1K).

**Figure 2:**
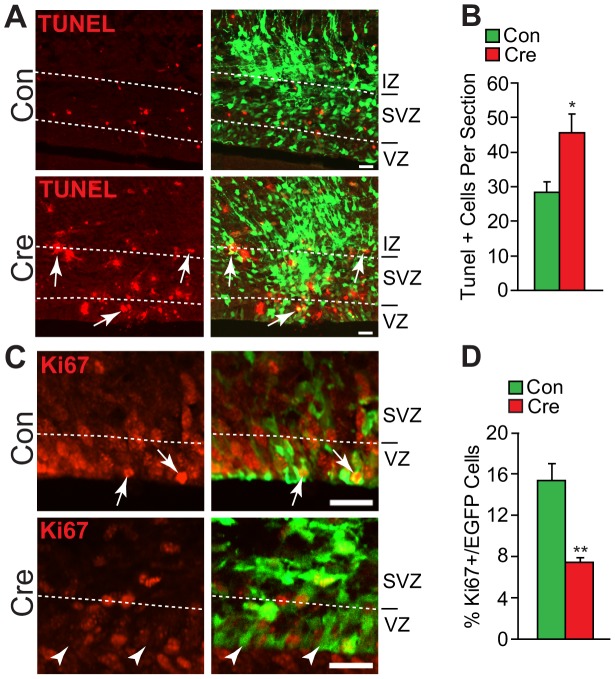
Snail controls cell survival and proliferation of cortical precursors in the embryonic cortex. Cortices of *Snail^fl/fl^* mice were electroporated at E13/14 with an expression construct that encodes both Cre recombinase and EGFP (Cre), and were analyzed three days later. As a control, cortices were electroporated with the same construct that encodes EGFP alone (Con). (A) Confocal micrographs of coronal cortical sections immunostained for EGFP (green) and labeled for TUNEL (red). The left panels show TUNEL alone, and the right the merges. The hatched white lines denote the different cortical regions. Arrows denote the double-labeled cells. Scale bar 20 µm. (B) Quantification of sections as in A for total TUNEL positive cells per section. (*p<0.05; n = 4 brains each). (C) Confocal micrographs of coronal cortical sections immunostained for EGFP (green) and Ki67 (red). The left panels show Ki67 immunostaining alone and the right panels the merges. The hatched white lines indicate the border between the VZ and SVZ. Arrows denote double-labeled cells and arrowheads EGFP-positive cells that are Ki67-negative. Scale bar 20 µm. (D) Quantification of sections as in C for the relative proportion of EGFP-positive cells that are also positive for Ki67. (**p<0.01; n = 4 brains each).

These data show that acute genetic ablation of *Snail* in radial precursors caused a decrease in precursors and neurons in the embryonic cortex, as we saw previously with shRNA-mediated knockdown [20]. In those studies, the decrease in cell number was due to an increase in cell death and a decrease in precursor proliferation. We therefore asked about both of these parameters in coronal cortical sections of *Snail^fl/fl^* embryos three days following electroporation at E13/14 with Cre-recombinase. TUNEL-labeling (Figure 2A) showed that there was a significant increase in the number of apoptotic cells in cortices electroporated with Cre-recombinase relative to controls (Figure 2B). Moreover, immunostaining for Ki67, a marker for proliferating cells (Figure 2C) showed that proliferating, Ki67-positive, EGFP-positive cells were reduced by more than two-fold following electroporation with Cre-recombinase, as compared to controls (Figure 2D). Thus, acute Cre-mediated genetic ablation of Snail in cortical radial precursors of *Snail^fl/fl^* embryos phenocopies results obtained with shRNA-mediated Snail knockdown [21].

### Genetic ablation of Snail in embryonic neural precursors causes perturbations in neural precursor proliferation in the neonatal cortex

**Figure 3:**
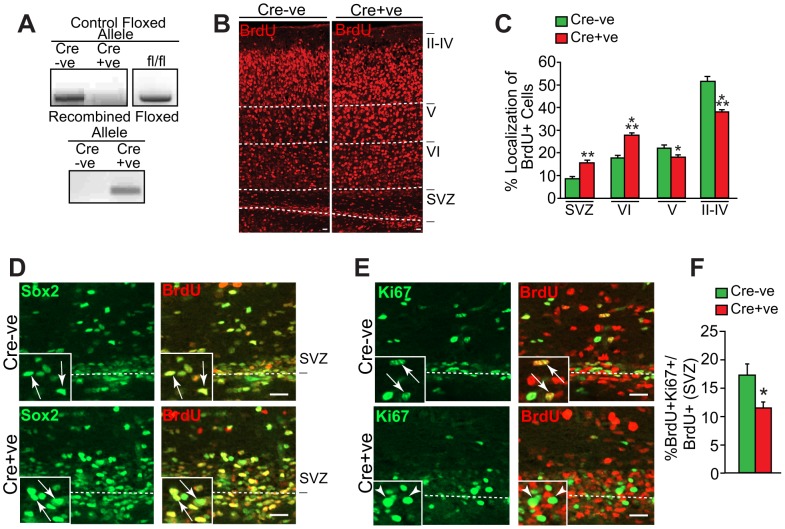
Perturbations in postnatal cortical precursors in Snail^fl/fl^;nestin-cre mice. (A) PCR analysis of genomic DNA isolated from the cortex of P7 *Snail^fl/fl^* mice that were positive (Cre+ve) or negative (Cre-ve) for the *nestin-cre* transgene showing the 480 nucleotide product from the intact floxed allele in the top panel, and the 492 nucleotide product generated from the floxed allele after Cre-mediated recombination (bottom panel). The top panel also shows genomic DNA from an adult *Snail^fl/fl^* mouse as a positive control. (B–F) *Snail^fl/fl^* mice that were positive (Cre+ve) or negative (Cre-ve) for the *nestin-cre* transgene were labelled with BrdU at E13 and their cortices were analyzed at P7. (B) Fluorescence micrographs of coronal cortical sections immunostained for BrdU (red). The dotted white lines demarcate the different cortical layers and the subventricular zone (SVZ), which comprises the precursor region in the postnatal cortex. Scale bar, 20 µm. (C) Quantification of sections similar to those in B for the relative location of BrdU-positive cells. (*p<0.05, **p<0.01, ***p<0.001; n = 3 and 4 *Snail^fl/fl^* mice Cre+ve and Cre-ve, respectively). (D) Confocal micrographs of the SVZ immunostained for BrdU (red) and Sox2 (green). The panels on the left show Sox2 alone and those on the right show the merges. The boxed insets show labelled cells at higher magnification, and the arrows denote double-labelled cells. Scale bar, 20 µm. (E) Confocal micrographs of the SVZ immunostained for BrdU (red) and Ki67 (green). The panels on the left show Ki67 alone and those on the right show the merges. The boxed insets show labelled cells at higher magnification. The arrows denote double labeled cells and arrowheads Ki67-negative, BrdU-positive cells. Scale bar, 20 µm. (F) Quantification of sections as in E for the proportion of BrdU-positive cells in the SVZ that are also Ki67-positive. (*p<0.05; n = 3 and 4 *Snail^fl/fl^* mice Cre+ve and Cre-ve, respectively).

We therefore used the *snail^fl/fl^* mice to ask about a potential role for Snail in the postnatal and adult cortex. To do this, we crossed them to a widely-used *Nestin-Cre* transgenic mouse line that drives Cre-recombinase expression in embryonic neural precursors, including those in the cortex [24]. Previous studies with this mouse line have shown that Cre-mediated recombination of a floxed reporter gene starts in the cortex at approximately E14.5, and that by postnatal ages, there is recombination of the reporter gene in the vast majority of cortical cells [31]. To ensure that the Snail locus was efficiently recombined in these *Snail^fl/fl^;nestin-cre* mice we performed PCR for the control floxed and recombined floxed alleles on DNA from the postnatal day 7 (P7) cortex. This analysis showed virtually complete recombination of the floxed *Snail* allele (Figure 3A), as predicted for this Cre driver line.

Having established the efficacy of this model, we asked if Snail was important for development of postnatal cortical neurons by crossing female S*nail^fl/fl^* mice with male *Snail^fl/fl^;nestin-cre* mice, injecting the pregnant mothers with BrdU at E13, and then analyzing their *Snail^fl/fl^* P7 pups that were positive and negative for the *Nestin-Cre* transgene. Immunostaining of coronal cortical sections from *Snail^fl/fl^* mice negative for the *nestin-cre* transgene showed that the majority of BrdU-positive cells were located in layers II-IV (Figure 3B,C). In contrast, in *Snail^fl/fl^* mice positive for Cre recombinase, there were proportionately more cells in layer VI and in the subventricular precursor zone (SVZ) (Figure 3B,C).

To ask if the increased BrdU-positive cells in the SVZ reflected perturbations in postnatal precursors, we immunostained sections for BrdU and either Sox2 or Ki67 (Figure 3D,E). Almost all of the BrdU-positive cells in the SVZ of both genotypes were positive for Sox2 (*Snail^fl/fl^;nestin-cre*, 97.8±0.49; *Snail^fl/fl^*, 96.6±0.38; n = 3 each). However, while approximately 18% of BrdU-positive cells in the control SVZ were Ki67-positive, this number was significantly decreased in *Snail^fl/fl^;nestin-cre* cortices (Figure 3F). Thus, as observed in the embryonic cortex, neural precursor proliferation was reduced in the neonatal SVZ by loss of Snail.

**Figure 4:**
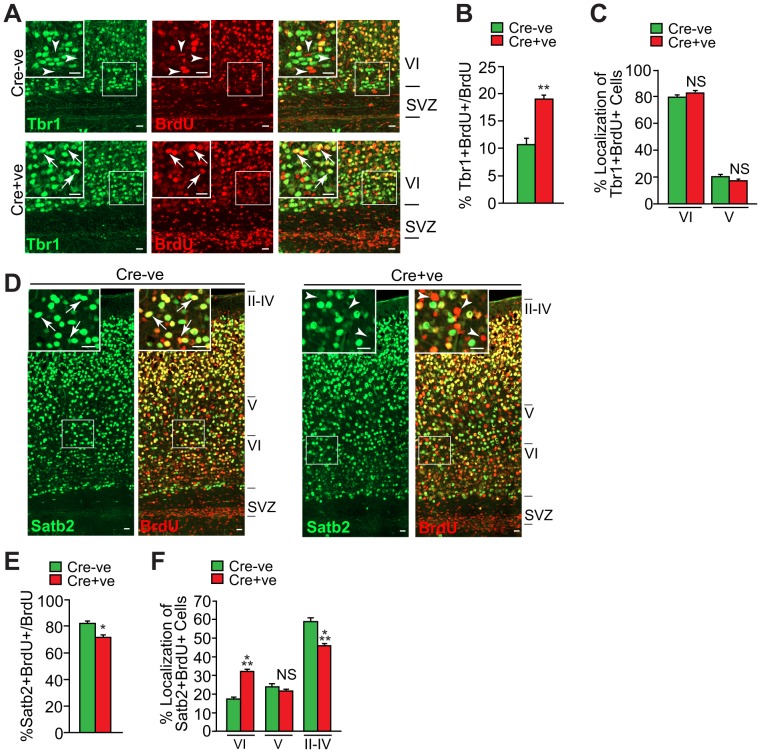
Postnatal deficits in cortical neurons in Snail^fl/fl^;nestin-cre mice. *Snail^fl/fl^* mice that were positive (Cre+ve) or negative (Cre-ve) for the *nestin-cre* transgene were labelled with BrdU at E13 and their cortices were analyzed at P7. (A) Confocal micrographs of coronal sections through the SVZ and layer VI immunostained for BrdU (red) and Tbr1 (green; merges are shown on the right). The boxed areas are shown at higher magnification in the insets. Arrows denote double-labelled cells. Scale bar, 20 µm. (B) Quantification of sections as in A for the proportion of BrdU-positive cells that are also positive for Tbr1 (B) and their relative location within layers V and VI (C). (**p<0.01, n = 3 and 4 *Snail^fl/fl^* mice Cre+ve and Cre-ve, respectively). (D) Confocal micrographs of coronal sections through the cortex immunostained for BrdU (red) and Satb2 (green). The left panels of each pair show Satb2 alone, and the right panels the merges. The boxed areas are shown at higher magnification in the insets. The arrows denote double-labelled cells. Scale bar, 20 µm. (E,F) Quantification of sections as in D for the proportion of BrdU-positive cells that are also positive for Satb2 (E) and their relative location within the different cortical layers (F). (*p<0.05, ***p<0.001, n = 3 and 4 *Snail^fl/fl^* mice Cre+ve and Cre-ve, respectively).

To ask if postnatal cortical neurons were also perturbed, as suggested by the localization data (Figure 3C), we immunostained similar sections with the layer VI neuronal marker Tbr1, which labels early-born neurons in the cortex (Figure 4A). In controls, approximately 10% of BrdU-positive cells were Tbr1-positive, while in *Snail^fl/fl^;nestin-cre* cortices, this number was almost doubled (Figure 4B). These Tbr1-positive cells were, however, appropriately localized in layer VI (Figure 4C). To ask if perturbations existed in later born neuronal populations, similar sections were immunostained for the layer II-IV marker Satb2 (Figure 4D). In controls, 80% of the BrdU-positive cells were Satb2-positive, whereas in *Snail^fl/fl^;nestin-cre* cortices there was a modest but significant decrease in this population of neurons (Figure 4E). Moreover, in controls these neurons were mainly localized to layers II-IV, but in *Snail^fl/fl^;nestin-cre* cortices, there were significantly fewer in layers II-IV and significantly more in layer VI (Figure 4F). Thus, genetic ablation of Snail caused both misspecification and mislocalization of cortical neurons.

### Genetic ablation of Snail perturbs adult forebrain neural precursor cells

**Figure 5:**
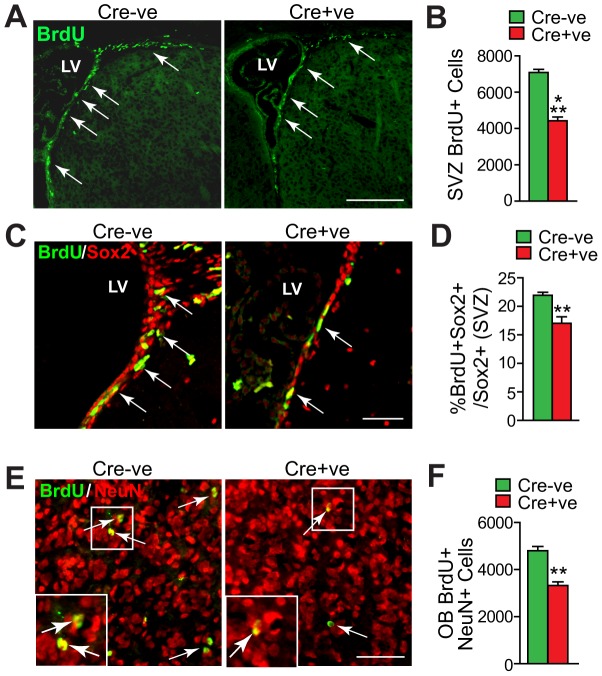
Genetic ablation of Snail perturbs the proliferation of adult forebrain neural precursor cells and the number of adult-born olfactory neurons. (A-D) Adult *Snail^fl/fl^* mice that were positive (Cre+ve) or negative (Cre-ve) for the *nestin-cre* transgene were injected with BrdU and coronal sections through the SVZ were analyzed one day later. (A) Fluorescence micrographs of sections through the SVZ that were immunostained for BrdU (green). Arrows denote BrdU-positive cells in the SVZ and rostral migratory stream. LV indicates the lateral ventricle. Scale bar 100 µm. (B) Quantification of sections as in A for the total relative number of BrdU-positive cells in the SVZ, as determined by counting all positive cells in 10 sections spanning the lateral ventricle (see experimental methods for details). (***p<0.01, n = 4 each). (C) Representative micrographs of coronal sections through the SVZ immunostained for BrdU (green) and Sox2 (red). Arrows denote double-labelled cells and LV indicates the lateral ventricle. Scale bar 50 µm. (D) Quantification of sections as in C for the proportion of Sox2-positive SVZ cells that were also positive for BrdU. (**p<0.01; n = 4 each). (E,F) One month old *Snail^fl/fl^* mice that were positive (Cre+ve) or negative (Cre-ve) for the *nestin-cre* transgene were injected multiple times with BrdU over the course of one day and the olfactory bulb was analyzed one month later. (E) Representative fluorescence micrographs of coronal sections through the olfactory bulb immunostained for BrdU (green) and NeuN (red). The boxed areas are shown at higher magnification in the insets, and arrows denote double-labelled cells. (F) Quantification of sections as in E for the total relative number of BrdU-positive, NeuN-positive cells in the olfactory bulb, as determined by counting all double-positive cells in 10 sections spanning the entirety of the olfactory bulb (see experimental methods for details). (**p<0.01; n = 3 each).

Since Snail is necessary for appropriate proliferation and numbers of both embryonic and postnatal precursors, we asked whether it was also necessary for adult forebrain neural precursor cells (NPCs) in the SVZ. Adult *Snail^fl/fl^;nestin-cre* and *Snail^fl/fl^* mice were injected with BrdU and analyzed one day later to determine the proportion of proliferating BrdU-positive cells in the coronal sections through the SVZ (Figure 5A). *Snail^fl/fl^;nestin-cre* positive mice had significantly fewer BrdU-positive SVZ cells than did *Snail^fl/fl^* controls (Figure 5B). To confirm that this reflected a decrease in proliferating NPCs, we costained similar sections with Sox2 and BrdU (Figure 5C). A significantly lower proportion of Sox2-positive NPCs were BrdU-positive in the SVZ of *Snail^fl/fl^;nestin-cre* versus *Snail^fl/fl^* mice (Figure 5D). To ask whether this decrease in proliferating NPCs led to a concomitant alteration in the number of adult-born olfactory neurons, we injected adult mice multiple times over the course of one day with BrdU and then, one month later, immunostained coronal olfactory bulb sections for BrdU and the pan-neuronal marker NeuN (Figure 5E). In both *Snail^fl/fl^;nestin-cre* and *Snail^fl/fl^* mice the BrdU-positive, NeuN-positive double-labelled cells represented a small proportion of the total neurons in the olfactory bulb (Figure 5E). Moreover, these adult-born neurons were distributed similarly throughout the extent of the olfactory bulb in both genotypes. In spite of these similarities, quantification showed that there were roughly 1.5-fold fewer adult-born olfactory bulb neurons in *Snail^fl/fl^;nestin-cre* versus *Snail^fl/fl^* mice (Figure 5F). To ask if this decreased neuron number was due to a specific decrease in neuronal differentiation or migration to the olfactory bulb, as opposed to the decreased number of NPCs, we normalized the numbers of adult-born neurons to the numbers of BrdU-positive SVZ precursors (Figure 5B). This analysis demonstrated that the number of olfactory bulb neurons per proliferating precursor was similar in both genotypes (*Snail^fl/fl^* 70%+/−3% versus *Snail^fl/fl^;nestin-cre* 77%+/−5%, p>0.05, n = 3 each). Thus, loss of Snail caused a decrease in the proliferation of forebrain NPCs, and a concomitant decrease in the numbers of adult-born olfactory neurons.

### Snail is also necessary for adult NPCs and adult-born neurons in the hippocampus

**Figure 6:**
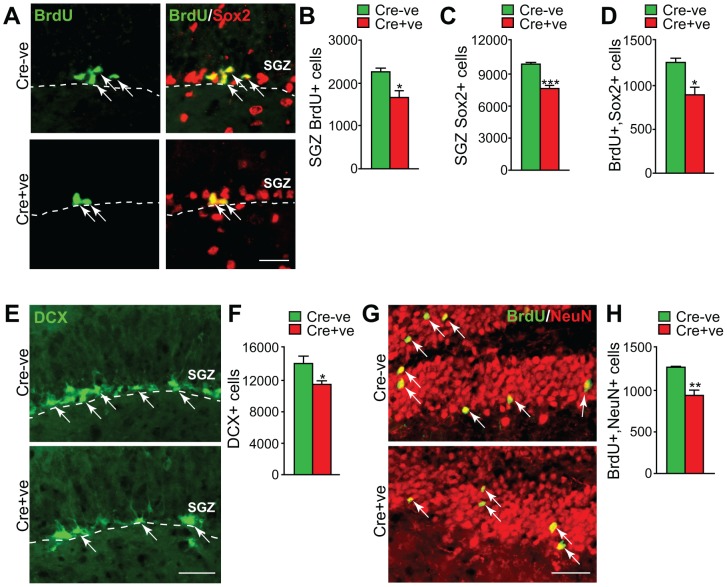
Genetic ablation of Snail perturbs the proliferation of adult dentate gyrus neural precursor cells and the number of adult-born hippocampal neurons. (A–D) Adult *Snail^fl/fl^* mice that were positive (Cre+ve) or negative (Cre-ve) for the *nestin-cre* transgene were injected with BrdU and coronal sections through the hippocampal dentate gyrus were analyzed one day later. (A) Fluorescence micrographs of sections through the dentate gyrus subgranular zone (SGZ) immunostained for BrdU (green) and Sox2 (red; right panels show the merged images). Arrows denote double labeled cells and the hatched white line the border of the SGZ and the hilus. Scale bar 50 µm. (B–D) Quantification of sections as in A for the total relative numbers of BrdU-positive cells (B), Sox2-positive cells (C) and BrdU-positive, Sox2-positive double-labelled cells (D) in the SGZ as determined by counting all positive cells in 10 sections spanning the dentate gyrus. (*p<0.05, ***p<0.001, n = 4 each). (E–H) One month old *Snail^fl/fl^* mice that were positive (Cre+ve) or negative (Cre-ve) for the *nestin-cre* transgene were injected multiple times with BrdU over the course of one day and the hippocampus was analyzed one month later. (E) Fluorescence micrographs of coronal sections through the dentate gyrus immunostained for doublecortin (DCX, green). Arrows denote doublecortin-positive cells and white hatched lines the border of the SGZ. Scale bar 50 µm. (F) Quantification of sections as in E for the total relative number of doublecortin-positive cells as determined by counting all positive cells in 10 sections spanning the dentate gyrus. (*p<0.05, n = 4 each). (G) Fluorescence micrographs of coronal sections through the dentate gyrus immunostained for BrdU (green) and NeuN (red). Arrows denote double-labelled cells. Scale bar 50 µm (H) Quantification of sections as in G for the total relative number of double labeled BrdU-positive, NeuN-positive cells, as determined by counting all positive cells in 10 sections spanning the dentate gyrus. (**p<0.01, n = 4 each).

To ask whether Snail is generally important for adult NPCs, we examined the other major NPC niche in the adult mammalian brain, the subgranular zone (SGZ) of the dentate gyrus. Adult 2 month old *Snail^fl/fl^;nestin-cre* and *Snail^fl/fl^* mice were injected with BrdU and were then analyzed 24 hours later. Immunostaining of coronal hippocampal sections with BrdU revealed that relative to *Snail^fl/fl^* mice, there were significantly fewer BrdU-positive cells in the SGZ of *Snail^fl/fl^;nestin-cre* mice (Figure 6A,B). Moreover, when we double-labelled similar sections for BrdU and Sox2, we found that the total relative number of Sox2-positive NPCs was reduced (Figure 6A,C), as was the total relative number of BrdU-positive, proliferating NPCs (Figure 6A,D). Thus, genetic ablation of Snail led to a decrease in proliferation and numbers of dentate gyrus NPCs.

To ask whether this decrease in *Snail^fl/fl^;nestin-cre* SGZ precursors was coincident with a decrease in the number of adult-born dentate gyrus neurons, we performed two assays. First, we quantified the number of SGZ cells expressing doublecortin, a marker for neuroblasts/newborn neurons (Figure 6E). Quantification throughout the extent of the dentate gyrus demonstrated a significant decrease in the relative number of doublecortin-positive cells in the *Snail^fl/fl^;nestin-cre* SGZ (Figure 6F). Second, to assay more mature adult-born neurons, BrdU was administered to 1 month old adult mice and then these mice were analyzed 30 days later by immunostaining for BrdU and the neuronal marker NeuN (Figure 6G). Quantification of the number of NeuN positive neurons that were BrdU-positive in the dentate gyrus (DG) showed that while these double-labelled neurons were similarly localized in both genotypes, the loss of Snail significantly decreased their numbers (Figure 6H). To ask if this decreased neuron number was due to a specific decrease in neuronal differentiation as opposed to the decreased number of NPCs, we normalized the numbers of adult-born neurons to the numbers of BrdU-positive SGZ precursors. This analysis demonstrated that the number of dentate gyrus neurons per proliferating precursor was similar in both genotypes (*Snail^fl/fl^* 56%+/−3% versus *Snail^fl/fl^;nestin-cre* 56%+/−3%, p>0.05, n = 3 each). Thus, Snail is generally important for regulating the number of adult NPCs and thereby determining the appropriate numbers of adult-born neurons.

## Discussion

The molecular mechanisms that regulate the number of neural precursor cells in the developing and adult brain are just now being elucidated. Data presented here address these mechanisms, and in so doing, define a role for the transcription factor Snail in neural precursor biology throughout life. In particular, work reported here using a conditional Snail knockout mouse model supports three major conclusions. First, data obtained with this mouse model confirms our previous acute shRNA-mediated Snail knockdowns [20], and show that acute ablation of *Snail* in the embryonic cortex altered the survival, proliferation and number of embryonic cortical precursors. Second, our data indicate that genetic loss of Snail in the postnatal brain caused deficits in postnatal cortical precursor proliferation, and neuronal perturbations, including an increase in cortical layer VI Tbr1-positive excitatory neurons, and a coincident decrease in, and mislocalization of Satb2-positive excitatory neurons. Third, our data indicate that Snail plays a similar role in adult NPCs. Specifically, in both the forebrain SVZ and the hippocampal SGZ, genetic ablation of *Snail* caused decreased precursor proliferation and numbers, and decreased numbers of adult-born olfactory bulb and dentate gyrus neurons. These findings indicate that Snail plays a general role in regulating neural precursor numbers throughout life, and provide support for the idea that many neural precursor regulatory mechanisms are evolutionarily conserved from model organisms like *Drosophila* through to mammals.

One major conclusion of this work is that Snail regulates neural precursor numbers throughout life by regulating their proliferation. This conclusion adds to an increasing body of evidence suggesting that many of the molecular mechanisms that regulate embryonic radial precursors also regulate their adult SVZ neural stem cell progeny (for example, see [3], [4], [6]–[8]). How then does Snail do this? Clues to the underlying mechanisms come from our recent work knocking down *Snail* in embryonic radial precursors using an acute shRNA-mediated approach [21]. In this study we showed, as observed here in the embryonic *Snail^fl/fl^* cortex, that when Snail levels were decreased, this caused increased death of cortical precursors, a robust decrease in radial precursor proliferation, and a resultant decrease in all of the cell types in the embryonic cortex. That study also showed that Snail regulates embryonic radial precursor survival via the tumor suppressor p53 and proliferation via the cell cycle phosphatase cdc25b. We posit that Snail likely regulates adult neural precursor biology via the same targets, since p53 has previously been implicated in adult SVZ neural precursor survival and proliferation [8], [32], [33], and cdc25 has previously been shown to regulate the cell cycle in adult dentate gyrus precursors under pathological conditions [34].

In addition to these neural precursor phenotypes, we also document robust neuronal phenotypes following genetic ablation of Snail, including neuronal mislocalization, neuronal misspecification and decreased neuronal numbers. With regard to neuronal mislocalization, we have confirmed our previous data showing that newborn embryonic neurons were aberrantly localized following acute genetic ablation of Snail [21]. More importantly, the BrdU birthdating studies in *Snail^fl/fl^* mice that are presented here show that Satb2-positive neurons born at or after E13/14 were mispositioned in deeper cortical layers at P7 in the *Snail^fl/fl^;nestin-Cre* mice. At both of these developmental timepoints, Satb2-positive neurons were located closer to the SVZ than they would be normally, suggesting that their migration was in some way impaired. This aberrant neuronal localization suggests a role for Snail in neuronal migration, a role it plays in other cell types [35]. Intriguingly, a recent study showed that the Snail superfamily member Scratch regulates delamination and migration of embryonic cortical neurons by transcriptionally regulating expression of E-cadherin [22]. Since Snail also regulates E-cadherin transcription [36], then this would provide a potential molecular mechanism to explain this phenotype. However, this interpretation is complicated by the fact that newborn cortical neurons migrate along the basal processes of radial precursor cells, and radial precursors are also perturbed when Snail is genetically-ablated.

With regard to neuronal misspecification, in the postnatal cortex of *Snail^fl/fl^;nestin-cre* mice the proportion of Tbr1-positive deep layer neurons was increased from 10 to 20%, while Satb2-positive neurons were decreased by a corresponding 10%. What is the explanation for this misspecification? One potential explanation is that newborn cortical neurons became misspecified as a consequence of their mislocalization. Precedent for this model comes from embryonic studies showing that when developing cortical precursors/neurons were transplanted into inappropriate cortical layers, they had the capacity to adopt aspects of the neuronal phenotype appropriate to their new location (for example, see [37], [38]). Thus, in our study, if neurons that would normally become upper-layer Satb2-positive neurons ended up instead in layer VI because they did not migrate appropriately, then perhaps they would adopt a layer VI Tbr1-positive neuronal phenotype, thereby explaining the increase in one and concomitant decrease in the other. Alternatively, this misspecification could be caused by a Snail-regulated shift in the ratio of radial precursors to intermediate progenitors. In particular, data presented here and in our previous study show that decreased levels of Snail cause a preferential decrease in the number of intermediate progenitors, thereby potentially leading to an increased relative proportion of radial precursors. Since deep layer neurons are predominantly generated directly by radial precursors while later-born, more superficial neurons are largely generated by intermediate progenitors [39], then a relative increase in radial precursors might result in an increased relative proportion of deeper-layer neurons, as we report here.

One final neuronal phenotype that occurred when *Snail* was genetically ablated was a decreased number of adult-born neurons. A key question is whether this is a consequence of deficits in adult neural precursors, or whether it might also be due to a requirement for Snail in the neurons themselves. In our previous study we showed that *Snail* knockdown led to death of both precursors and neurons in the embryonic cortex, and that this occurred in a p53-dependent fashion. Since p53 activation can cause apoptosis of adult-born neurons in a PUMA-dependent fashion [8], and since the Snail family can repress an apoptotic p53-PUMA-dependent pathway in other mammalian cell types [40], [41], then it is possible that Snail is important for survival of newborn neurons, whether they are generated in the adult or in the embryo. However, defining a direct effect on neuronal survival will require manipulation of Snail in a neuron-specific fashion.

In summary, our findings define a role for Snail in the regulation of mammalian neural precursor biology throughout life. In all of these different developmental contexts, Snail regulates the proliferation and number of neural precursors, and coincidently determines the number, positioning and phenotype of newborn neurons. These findings define a transcriptional regulator that plays a key role in mammalian neural development, and contribute to the growing concept that many of the molecular factors that control behavior of neural stem cells are conserved from *Drosophila* to mammals [11]–[14].
